# Sustainable poverty amelioration through early life education in a peri-urban community of Lagos, Nigeria

**DOI:** 10.4102/phcfm.v2i1.98

**Published:** 2010-06-14

**Authors:** Olayinka A. Abosede, Princess C. Campbell, Emmanuel I. Okechukwu, Ajibike O. Salako-Akande, Anthonia O. Onyenwenyi

**Affiliations:** 1 Institute of Child Health and Primary Care, University of Lagos, Nigeria; 2 Action Family Foundation, Lagos, Nigeria; 3 WELLCHILD Promotion Organization of Nigeria, Lagos, Nigeria

**Keywords:** early life education, economic empowerment, Nigeria, peri-urban community, poverty amelioration

## Abstract

**Background:**

Daycare centres/nurseries have become popular because of the need for working mothers to leave young children with caregivers. However, the high poverty level (54% relative and 35% extreme poverty) makes it difficult for disadvantaged parents to pay the high fees charged by the centres. This study describes an attempt to economically empower mothers through the organisation of free early life education in a peri-urban community in Lagos.

**Objectives:**

The aim of the study was to examine early life education for under-fives as a means of economic empowerment of mothers and sustainable poverty amelioration.

**Method:**

The methodology included a non-randomised selection of 34 disadvantaged mothers by criteria, a prospective intervention utilising community resources to organise early childhood education, an in-depth interview of mothers, and observation of the outcomes over a 5-year period.

**Results:**

The result of the study showed that no mother preferred keeping a child older than three years at home. Access to early childhood education gave mothers opportunity to undergo vocational training (1, 2.8%) and take up new/additional jobs (12, 35.3%). All mothers and 32 (80%) of the participating families more than doubled their income, earning up to twenty thousand Naira (approximately $182) per month from the first year of participation. Finally, selection criteria and periodic assessment of immunisation/growth monitoring records of participants’ children improved compliance with primary health care service utilisation.

**Conclusion:**

Organisation of early childhood education had the potential for sustainable poverty amelioration through economic empowerment of mothers.

## INTRODUCTION

Poverty is defined by an encyclopaedia as ‘the condition of not having the means to afford basic human needs such as clean water, nutrition, health care, education, clothing and shelter’.^1^ All of these determine our quality of life. It may also include the lack of access to opportunities such as education and employment, which aids the escape from poverty and/or allows one to enjoy the respect of fellow citizens. Nigeria is the most populous African country (140 million people in 2006)^2^ and in Lagos, the former capital popularly known as the country's ‘economic nerve centre’, many people live below the international poverty line (earning less than $1 per day). Despite Nigeria's oil exports, the country's poverty rate is high, about 60%,^3^ or 54% for people living in relative poverty and 35% in extreme poverty.^4^

The middle class is almost non-existent, making the gap between the rich and the poor very obvious.^4^ While many children of middle- to high-income families are already in ‘early life education’ programmes, children of low-income families are more likely to be seen playing during the day, sometimes with no adult supervision. One hears, from time to time, of a mother looking for her child, or tragic stories of accidents (like drowning in an uncovered well) in the study community. However, in spite of their poverty and struggle to survive, research has shown that the education of children is a priority to many of these families.^5^ Even those living in the poorest of areas want their children to have sound education early in life and would rather go without meals in order to send their children to fee-paying private schools. According to a study by James Tooley, about 65% to 70% of schoolchildren from the poor areas were in private primary schools.^6^ In a study in Lagos, a fisherman noted that:

‘*In the public school they do not teach very well and even though we are very poor we prefer to send our children to private schools because we want our children trained for the future’*.

Similarly, a mother from that same study made the following comment about private schools:

*‘In the private school they teach them very well, they study very well and the students are sharp. That's why I prefer to send my children to private school’*.

There are several kindergarten/daycare/nursery schools attached to primary schools in Lagos,^7, 8^ but many of them, through self-help initiatives, are profit-oriented and not accessible to poor families.

There is a vicious cycle between poverty, literacy and illness and so economically disadvantaged families are more likely to fall ill, causing high morbidity and mortality rates in their communities. Also, a mother's level of education and economic power affects the health status of her family and, in particular, her own health and that of her children under five years old. Better educational achievement, increased chances of employment and earning power have a positive influence on the health knowledge, attitude and behaviour of mothers who are not only care-givers but who are increasingly becoming providers of the basic needs of their children.^9^

Nigeria is one of the countries with the lowest coverage for immunisation and growth monitoring and this has resulted in its high under-five morbidity and mortality rate (250 per 10 000), in spite of appropriate government policies and various attempts to improve service quality and utilisation.^10, 11^ Community ownership of health and development programmes, even though emphasised in the National Health Policy, is not fully embraced by the stakeholders and implementers of the policy.^12^

The Ward Health System was adopted in Nigeria in the 2000. A ward is the smallest geopolitical administrative entity from which a councillor is elected to the next level of government unit, the local government authority (LGA). It may comprise several villages or groups of communities, each usually having its own development committee. Each ward has a total population of about 10 000–30 000. There are officially 8812 wards in Nigeria and 375 in Lagos state. Ward development committees (WDCs) or community development associations (CDAs) are community organs that have responsibilities for ensuring the implementation of health and developmental interventions.^13^

## ETHICAL APPROVAL, PERMISSIONS AND INFORMED CONSENT

Ethical approval was obtained from the Ethics Committee of the College of Medicine, University of Lagos and permission to collect data in the schools and communities was obtained from the Lagos State Universal Basic Education Board and the Ikosi-Isheri local council development area (LCDA) respectively.

In view of the apparent sensitivity of the mothers to social status issues, potential participants were individually interviewed to ascertain their willingness to participate in the study. Informed consent of the fathers (husbands of participants) was also obtained directly or through their wives.

## METHOD

### Advocacy

The concept of the 5-year prospective community-based intervention was explained to 18 members of the CDAs of Magodo, the study community. Members of the neighbouring communities of Isheri (22), Olowora (26) and Omole Phase 2 (32) were also addressed during monthly meetings. Influential community members of 15 Residents’ Associations (RAs) in the high socio-economic bracket were also paid advocacy visits during monthly meetings in the four communities. Emphasis was laid on the need to utilise resources available within the ward to assist poor, disadvantaged families.

Support in form of sponsorships from the proprietors of seven private nursery and primary schools in the ward was solicited. Sponsorship was not required until the children of study participants attained the age of 5 years. The number of pupils sponsored was determined by each school.

### Selection of participants

The participating mothers had to comply with the following selection criteria:

- Either they were single parents (e.g. widow), or if they were married, they and/or their husbands worked within the community as artisans.
- They worked away from home.
- They had no one else to take care of an under-five child.
- They were of low socio-economic status, earning less than the basic minimum wage of five thousand Naira (approximately $45 per month); their total family income had to be less than twenty thousand Naira ($182 per month).
- They were willing to undertake vocational training within the community if necessary.
- They were willing to enrol their children for early life education.
- They presented the child's birth certificate, immunisation record and growth chart.

#### Daycare/early childhood education

One of this study's key intervention strategies was to increase the enrolment of children under 5 years old in school, so that the mothers were free to engage in economic activities. WELLCHILD, a community-based non-governmental organisation provided the necessary space in a homely environment for the daycare/ early childhood education programme. It sourced for and engaged volunteer teachers that included mothers and youths from the community/ward. Initial educational materials and standard syllabi were obtained from a private school in the ward and training materials were donated by friends of the organisation. The mothers were encouraged to bring their children to the school at 08:00 and to fetch them at 12:00.

The maximum number of children at any time was restricted to 10 to maintain high quality teacher–pupil interactions. The first set of six children aged 3–4 years was taught as one class for the first year. They were joined by four more children before separation into two classes in the second year (three children in the 3–4 age group and seven in the 4–5 age group). The pupils conducted general activities (singing, praying, physical examination for hygiene and recreation) together daily, but were separated by age group for other, more specific learning activities.

The number of children who left for primary schools after the age of 5 years were replaced each time by those who had been waiting for enrolment. Five of the children were sponsored; four fully (two by the private schools and two by community members) and the fifth was partly sponsored (half-tuition) by a school. Two children in the 3–4 age group who left when their families moved to another zone in the community were also replaced. A total of 40 children were enrolled over the study period of 5 years (Table 1). A maximum of 10 children were allowed in the programme per year and they graduated to nursery 2/primary school when they reached 5 years old.

**TABLE 1:** Enrolment and graduation statistics for the WELLCHILD early life education programme, 2003–2008

Year	Age 3–4 years	Age 4–5 years	Graduated or moved	Total newly enrolled during the year
2004	6	0	0	6
2005	5	2	0 graduated, 3 moved and 2 replaced	7
2006	11	1	7 graduated, 2 moved and 2 replaced	12
2007	4	4	11	8
2008	4	3	8	7

**Total**	**30 (75%)**	**10 (25%)**	**31 (77.5%)**	**40 (100%)**

The programme was conducted in Magodo Ward A, Ikosi-Isheri local council development area, Lagos, Nigeria.

#### Sponsorship for primary education

Three categories of scholarship for primary education were solicited for pupils on completing the programme. These were, (1) full tuition waived by a private primary school, (2) half tuition waived by a government primary school and (3) all expenses to be paid to the school by an individual sponsor.

Only one child attended the government primary school because their older siblings attended the primary and secondary schools on the same premises.

#### Child health services

The children's birth certificates were used to verify their ages and their green cards (primary health care child health home-based records) were checked before registration for relevant information about the families and to assess immunisation status and regularity of growth monitoring.

None of the first set of children enrolled possessed a green card, was fully immunised or had their growth monitored. They, and all the children subsequently enrolled, had to possess two cards, one kept at the community's Health Post and one kept at home. The children were all fully immunised at registration and their growths were monitored using the primary health care growth chart on weight-for-age every other month.

#### In-depth interview of mothers

All mothers were interviewed at the beginning, at different times during and at the end of the study, with schedules that probed socio-economic activities (both theirs and/or their husbands’), obstacles and constraints to having gainful employment or improving on current work. Their claims of training and new/ additional jobs were verified by their teachers who were also resident in the community. Interviews were done when mothers came to collect their children. Participants were also interviewed on their awareness of the government programme that started in the neighbouring Isheri community within the ward a few months after the study commenced.

#### Counselling of mothers

Mothers were counselled on the types of employment available in the community, for example, those available in some houses/ streets (cleaning, cooking, clothes washing), at the local bakery, at factories (table ‘pure’ water) and at building sites (bricklaying, labour assistants for odd jobs that including fetching water, mixing cement, carrying cement mix to points of use etc.). Cleaning jobs were in the homes of more affluent community members, on streets and in some churches and mosques. Clothes washing and cooking on a weekly or monthly basis were common jobs in the University of Lagos zone, where residents were mostly retired university staff.

### Sourcing for volunteer teachers

Appeals for volunteer teachers were made during the initial advocacy visits. The teachers were recruited and given orientation on the study and child care. Categories of community residents who were screened and recruited included:

- volunteer mothers (Two at a time, and a total of four for the 5-year study period. All had at least secondary/high school education and one was a nursery school teacher who moved into Magodo earlier.)
- unemployed youths (One tertiary institution graduate who was also the Administrative Officer for WELLCHILD, two youths awaiting Joint Admissions Matriculation Board results to tertiary institutions, and one with a West Africa School Leaving Certificate for secondary school education.)
- two previous beneficiaries of scholarships for Ordinary level diplomas from WELLCHILD.

All the teachers consented to a token honorarium of two hundred Naira (approximately $1.80) per day for helping for 4 hours each day.

The first group of volunteer teachers for the first set included a single mother of two adult daughters, one of whom had learning difficulties. The mother worked in the WELLCHILD daycare in the morning and joined her daughter later in the day for petty trading. She possessed the West Africa School Leaving Certificate and served in the programme for 2 years, until she obtained space in a nearby market. In her place, a volunteer youth awaiting admission into a tertiary institution served for the next year. The first group also included a housewife with primary school education who, after having received counselling, taught in the school as an alternative to selling the local illicit gin known as *ogogoro*. On moving out of the community (after serving for 1 year) she was replaced by a youth who had benefited from a WELLCHILD scholarship; the scholarship had allowed him to complete the last year of his secondary education.

Teachers for the second set included two unemployed youths who served for 1 year, until they gained admission into tertiary institutions. They were replaced by the third set of two volunteer mothers, both of whom were petty traders with secondary/high school education. The child of one of these mothers benefited from the early life education programme.

### Limitations

Two limitations that were identified are, (1) the small sample size because of limited resources and the necessity for quality care and education (teacher-to-pupil ratio of 1:5) for participants’ children in the early life education programme and (2) the reliance on the sincerity of participants (e.g. for information on individual and family income).

## RESULTS

### Participant profiles

Of the 40 children enrolled during the study period, 21 (52.5%) were females and 19 (47.5%) males. As the children reached the age of 5 years and moved to Primary Schools, they were replaced to maintain a maximum of 10. The total number enrolled over the 5-year programme was 40 from 34 families. Thirty (75%) of the enrolled children entered the programme when they were aged 3–4 years. There were 26 (65%) graduates out of the 31 (77.5%) who had left the programme by the time of this report (Table 1).

Most of the mothers who participated in the study were Yoruba (20, 58.8%). Others were Egun early settlers (7, 20.6%), Urhobos and Edos (7, 20.6%). Most of them fell within the age group 25–45 years (32, 94.1%). There were 3 single parents (one teenage mother, a widow and one who had separated from her husband). One mother had 3 children under five years old and one had twins.

In total, 34 women were selected, 14 (41.2%) were full-time housewives, 8 (23.5%) were petty traders, while 4 (11.8%) were hairdressers, 3 (8.8%) street cleaners, and 3 (8.8%) vegetable/ fruit sellers and food vendors (Table 2).

**TABLE 2:** Family profile of participants

Family	No. of children under 5 years (*N* = 40)	Father's occupation	Mother's occupation
1	1	Not applicable (teenage mother/single parent)	Casual sachet water factory worker
2	1	Not applicable (separated/single parent)	Street cleaner
3	1	Welder	Street cleaner
4	1	Security guard	Petty trader
5	2	Bricklayer	Fruits seller
6	1	Not applicable (widow/single parent)	Vegetable seller
7	1	Commercial motorcycle	Housewife
8	1	Unemployed	Petty trading
9	2	Commercial motorcycle	Housewife
10	1	Casual labour jobs	Petty trader
11	1	Imam (Muslim Priest)	Tailoring
12	1	Carpenter	Housewife
13	1	Casual labour jobs	Housewife
14	2	Casual labour jobs	Housewife
15	1	Wood seller	Hairdresser
16	1	Imam (Muslim Priest)	Petty trader
17	1	Casual labour jobs	Petty trader
18	1	Teacher	Housewife
19	1	Casual labour jobs	Housewife
20	1	Casual labour jobs	Petty trader
21	3	Security guard	Food vendor
22	1	Bricklayer	Petty trader
23	1	Carpenter	Petty trader
24	1	Bricklayer	Housewife
25	1	Casual labour jobs	Hairdresser
26	1	Welder	Hairdresser
27	1	Mechanic	Hairdresser
28	1	Casual labour jobs	Housewife
29	2	Bricklayer	Street cleaner
30	1	Casual labour jobs	Housewife
31	1	Casual labour jobs	Housewife
32	1	Casual labour jobs	Housewife
33	1	Casual labour jobs	Housewife
34	1	Casual labour jobs	Housewife

The early life eduction programme was conducted in Magodo Ward A, Ikosi-Isheri local council development area, Lagos, Nigeria.

All families earned a combined income of less than ten thousand Naira (approximately $80) per month at the beginning of the study.

Of the 31 participants’ husbands, most, 13 (41.9%) were artisans who did unspecified casual labour jobs at construction sites in the Government Reservation Area carved out of the original Magodo community. There were also bricklayers (4, 12.9%), commercial motorcycle operators (2, 6.5%), security guards (2, 6.5%), welders (2, 6.5%), carpenters (2, 6.5%), Imams/Moslem priests (2, 6.5%) and others (4, 12.9%) (Table 2).

The early life education programme organised for the participants’ children enabled all of them to function better (Table 3) and 12 mothers (35.3%) took up new/additional jobs within the community during the first few months of the study, enabling them to improve their earning power. One mother had opportunity to train as a tailor and all other former full-time housewives started casual jobs. All (100%) of the 7 families that participated in the first year and approximately 80% of all participating families more than doubled their income or earned more than twenty thousand Naira (approximately $182) per month in the first year of participation.

**TABLE 3:** Empowerment of participating mothers

Newly acquired job and/or improved functionality and empowerment	No. of mothers (*N* = 34)	Proportion of mothers (%)
More freedom to complete house chores	15	44.1
More freedom to go and purchase wares to sell	10	29.4
Took up additional jobs (house cleaning and/or water fetching) in community	6*	17.6
Child sponsored for primary education	5	14.7
Better concentration in saloon	4	11.8
Started petty trading	3*	8.8
Employed as nursery school teacher/ teacher in WELLCHILD programme	2*	5.9
Full time as pure water factory worker	1	2.8
Started and completed apprenticeship in tailoring	1*	2.8

The early life education programme was conducted from 2003–2008 in Magodo Ward A, Ikosi-Isheri local council development area, Lagos, Nigeria.

* The total number of women who took up new or additional jobs was 12 (35.3%).

### Enrolment in other schools or primary schools

For comparative purposes, the levels of enrolment to the government-operated nursery school that was attached to the Isheri Primary School, as well as the United African Church Community (UACC) Nursery School within the same compound were also assessed (Table 4). Records were obtained from the data collated by the School Services section of the State Universal Basic Education Board and registers of the two schools.

**TABLE 4:** Enrolment at Isheri Government and UACC Nursery and Primary Schools, 2003–2008

Year	Isheri Public Nursery and Primary School	UACC Nursery and Primary School	Total
2003–2005	0	0	0
2006/2007	28	24	52
2007/2008	29	21	58

It was found that no child had enrolled in the government and UACC Nursery and Primary Schools, in Isheri between 2003 and 2005, while the enrolment totalled were only 52 and 58 respectively in the 2006/2007 and 2007/2008 sessions, respectively (two of these children were only 2 years old on enrolment).

From the beginning of the study, it was found that participants no longer kept a child above 3 years at home; all participants’ children were enrolled in schools throughout the study period. However, none of the first-year participants was informed when the government started a programme for under-fives attached to the primary school in Isheri, the immediate community within the ward. Even though all 34 participants were eventually informed about it by the WELLCHILD teachers, they did not wish to enrol their children there. The reasons given by all were the distance and the need to ride on commercial motorcycles or tricycles. Two participants (5.9%), after improvement in their income, voluntarily took responsibility for sending their children to nearby private fee-paying primary schools. Five of the seven children in the first set (71.3%), on attaining the age of 5 years, were sponsored for primary education in private schools within the ward, while two families paid fully in a nearby private school.

There was better guarantee that a child's education would not be disrupted if the school waived the tuition fee than if sponsored by residents in the ward. Two individual sponsors could not fulfil their obligations after the first year and WELLCHILD had to source for alternative sponsors.

## DISCUSSION

### Community leaders’ commitment to the programme

All the LCDAs and RAs were positive in their response to the concept of women empowerment through the organisation of the early life education programme. One CDA (Omole Phase 2) and one RA (Salako zone) committed funds to a private school's WELLCHILD s services charge for the shop operated by one of the school teachers to generate funds for the programme.

The expressed and demonstrated commitment of community leaders is one of the strengths of the early life education intervention for economic empowerment of mothers. Sustainability of the programme depends on their continued commitment and efforts to see that all under-fives have opportunities for enrolment in standard educational programmes thereby releasing their mothers for gainful activities. That the poorest of families would struggle to send their children to school, even private, fee-paying ones, in spite of their predicaments, has been confirmed by various studies in India, Ghana, Kenya and Nigeria.^5, 14, 15^ Discussions on the internet^7^ and in some newspapers^16, 17, 18, 19, 20^ have expressed the need to put equity, one of the basic principles of Primary Health Care, into practice.

LGAs and LCDAs, the government organs responsible for basic education up to primary level, need to identify all the self-help and private schools, whether registered or not, and absorb them into the national universal basic education programme. Since the performance of pupils, even in unregistered private schools, has been found to be better than in government schools^14^ it may be better for community development committees to organise educational programmes for their children, or to establish private–public partnerships on education with relevant ministries or responsible government organs. This way, the unacceptably high pupil-to-teacher ratios in government schools in Nigeria (and as found in Kenya and other developing countries^21^) can be reduced and better standards ensured. After comparing notes with children in private schools, parents in Ghana withdrew their children from free government schools and returned them to the fee-paying private ones. Wherever educational entrepreneurs, especially those in poor communities, set up schools to cater to unfulfilled demands, they should be supported by government and their efforts optimised.

### Economic empowerment

The primary expected outcome of this study was that participants would be able to utilise the better opportunities provided to them by enrolling their children in the early life education programme to engage in gainful activities (new/additional employment) that were previously limited. It is relatively easier to manage a baby less than 3 years old, who can be strapped to the back, than an inquisitive 3–5-year-old, even for those parents who are self employed. Additionally, where a mother has more than one child under-five, enrolling the older one in early life education provides her with a better opportunity to take care of the younger one (reducing predisposition to infections, malnutrition, home accidents, etc.) and to perform more efficiently in other, more gainful ventures.

Among Nigeria's efforts to reduce poverty are those undertaken by the National Poverty Eradication Programme, which include:

- the youth empowerment scheme
- the establishment of a DataBank for the unemployed
- the mandatory attachment/resettlement of graduates, tailoring and fashion designer trainees
- the Giveback Programme for Nigerians in the diaspora
- blindness prevention and treatment
- rural communication programmes
- the establishment of youth information centres
- the institution of the Capacity Acquisition Programme and the Village Economic Development Solutions Model.

It has been reported that the Nigerian government has released funds for the implementation of such programmes.^8^ When mothers are confident that their children are safe, they will be able to enrol in any of the programmes as appropriate. There are similar efforts in other developing countries like Kenya, Ghana and India that have been reporting successes.^8, 22, 23^

### Enrolment in other schools or primary schools

Many mothers may not be aware that free early life education programmes for children aged 3–5 years are attached to many government primary schools. Some may also not want to enrol their children in schools that are not within walking distance for the children as found in this study. In the study ward, road traffic accidents, especially from motorcycles, were the third most prevalent problem for 2006/2007,^24^ yet some parents and children rode on motorcycles without helmets. The study participants’ reluctance to send their under-fives to Isheri because of the distance (approximately 1 km – 3 km) and the dangers to which the children are exposed on the roads should be taken into consideration by government and community committees.

An earlier demographic study in Magodo reported a total population of 9308 and, of this, 1794 (19.3%) were under-fives.^24^ Of the latter, 1384 were more than 1 year old,^24^ yet the number of children who were enrolled in the Isheri government Nursery and Primary School and the UACC Nursery and Primary School (which enrols children 2 years or older) were significantly low, with only 52 and 58 enrolment in the 2006/2007 and 2007/2008 sessions, respectively. This indicates that under-fives were either in private schools, or not in any early education programme, in spite of the fact that the public schools were free. Major reasons for this may be lack of awareness and the distance of the schools from Magodo, as reported by participants in this study.

### Availability of government funding

One of the achievements of the education sector is ‘empowerment of communities to participate actively in the management and eventual ownership of schools through the Self-Help approach’.^25^ There is no doubt that community members need to be fully involved at all levels (planning, implementation, monitoring and evaluation) of early life education programmes for their communities. However, sometimes funds are made available by governments, but communities (the beneficiaries of such funds) are not aware and officials do not fulfil requirements to access such funds for years. An example of this would be the funds allocated by the Nigerian Federal Government for universal basic education, which is another laudable effort towards the attainment of the Millennium Development Goals (MDGs). According to the Executive Secretary of the Universal Basic Education Commission, to date, about one hundred and eleven billion Naira (approximately $94 000 000) has been allocated, but has not been accessed by 27 out of 36 states and the Federal Capital Territory since the 2005.^26^ Early childhood care and development education shares 5% of the funds, while primary education and junior secondary education receive 60% and 35% respectively. In order to access the funds, action plans are to be submitted by states and counterpart funds are required. Judicious and optimal utilisation of funds allocated for early childhood education, in addition to existing community resources, will accelerate the achievement of at least five of the MDGs, including poverty eradication, directly and/or indirectly. Even though the MDG on education is *‘*to ensure that boys and girls everywhere complete a full course of primary education by 2015’, many studies have found an early life education programme desirable and essential for maximising children's potentials.^27, 28, 29^ Also, advocates for *free* programmes believe in equity and the fact that ‘free early learning gives children the best start in life through learning and playing with other children in a safe and structured environment. They have a head start when they start school’. The advocates have emphasised the need for a national curricula framework and appropriate content for children aged from birth to 3 years, for children aged 3–6 years, as well as an improvement of the transition between early childhood education care settings and primary schooling.^30^

Children of poor families also deserve the right to a good start in life. Even when these education programmes are made free, high standards must be maintained and they should be within walking distance for the children. This, and the population of children under 5 years, indicate the need for more than one school within a community. Community-organised (private) schools should thus be assisted to complement government schools. Easy accessibility will motivate mothers to drop off their children in these programmes before going to work or the market.

Private fee-paying schools willing to provide tuition-free services to the less privileged in their communities should also receive government assistance. Even though 60% of the first set of children at WELLCHILD had sponsorship to nearby private primary schools, disappointment from individual sponsors in less than two years confirmed that there is a surer guarantee if the school waived the tuition fees than if sponsorship was provided by residents. Private schools, worldwide, are usually more expensive than government schools and often require additional costs to tuition. Individual sponsors may not pay regularly or stop altogether when they have diverted their funds to other priorities. Compliant private schools should thus be compensated for sponsorship, for example, through taxation reduction.

### Primary health services

The study showed that encouraging mothers to ensure that their children had birth certificates, received necessary immunisations and had regular growth monitoring is positive for improved coverage and service utilisation. The low national coverage of immunisation and growth monitoring, as well as service utilisation for other basic, yet important, health interventions, will be improved if there is a collaborative effort between the Ministries of Health and Education.

### Sustainability

The early life education programme organised by, and within, the community, involving community members (not teachers posted by government from other communities) attracted better patronage and was sustained through community contributions. It can be safely assumed that every ward in Nigeria has resources that can be harnessed for ameliorating or eradicating poverty of disadvantaged families. In order to promote self-reliance and sustainability, resources within communities/wards must be identified and relevant development committees established, with the WDCs, CDAs and RAs assisting in the utilisation of such resources equitably for the benefit of residents. If each of the 8812 wards in Nigeria operates an early life education programme, about 15–20 million mothers will have better opportunities for sustainable economic empowerment and poverty amelioration.

## RECOMMENDATIONS AND CONCLUSION

While acknowledging its limitations, this paper recommends free early life education to promote women's empowerment and poverty amelioration. This should be organised in communities that are more than 1 km from government schools. For quality to be maintained, teacher-to-pupil ratios and facilities must be acceptable to appropriate standards organisations. Also, easy accessibility may mean having more than one school for underfives in each community constituting a ward.

Poverty amelioration through economic empowerment of women has national and global relevance as well as direct/ indirect implications for attainment of the MDGs. This preliminary study, even though a small sample, has shown that beneficiaries (disadvantaged mothers and families) will benefit from free early childhood education programmes that allow them freedom to pursue economic ventures.
